# Nurses’ high valuation of palliative care versus patient and family misconceptions: A mixed-approach study of Advanced Care Planning implementation in China

**DOI:** 10.1371/journal.pone.0333739

**Published:** 2025-10-07

**Authors:** Chenyan Liang, Suzhen Lv, Jia Song, Leiwen Tang

**Affiliations:** 1 Department of Nursing, The Second Affiliated Hospital of Zhejiang University School of Medicine, Hangzhou, People's Republic of China; 2 Department of Rheumatology and Immunology, Lishui Central Hospital, Lishui, Zhejiang, China; Murcia University, Spain, SPAIN

## Abstract

**Objective:**

To assess the implementation and barriers of Advance Care Planning (ACP) from the perspectives of nurses, patients, and family members in a Chinese healthcare setting, providing evidence for targeted interventions to enhance ACP awareness.

**Methods:**

A mixed-methods design combined a cross-sectional survey and qualitative interviews. Questionnaires measured ACP-related knowledge, attitudes, practices (KAP), and perceived barriers among 843 nurses, 384 patients, and 857 family members. Based on responses, 15 patients and 20 family members with negative views on ACP were invited for semi-structured interviews and focus groups.

**Findings:**

Nurses rated ACP as highly important and reported effective palliative care practices, while patients and families showed substantial cognitive gaps. Over 90% of patients valued ACP, but only ~30% believed it was well implemented; many families associated it with treatment abandonment. Multivariable analysis identified hospital type, clinical experience, and information sources as KAP predictors. Qualitative results linked misconceptions to poor communication and insufficient public education.

**Conclusion:**

Nurses endorse ACP, but patients and families often misinterpret it as giving up treatment. Improved communication and tailored education are critical to increasing ACP acceptance in China.

## 1. Introduction

Advance Care Planning (ACP) refers to the process by which individuals, even when healthy, make decisions about future medical care in case of serious illness, and communicate those preferences to family and healthcare providers [1]. This process involves multiple stakeholders (patients, family, physicians, nurses) and settings, requiring communication and documentation that reflect one’s beliefs about death, cultural values, and support systems [2]. Palliative care addresses pain, symptoms, and psychosocial-spiritual needs, and often provides the supportive context in which ACP conversations occur. In essence, ACP can be seen as a component of palliative care planning: it enables patients to assert their care preferences in advance, thereby facilitating a course of treatment that respects their goals and improves end-of-life quality [3]. International experiences show wide variation in ACP implementation, with some countries achieving high engagement and clear benefits. In places like the United States, Canada, and Australia, ACP has been promoted through national policies and public campaigns for decades. For example, in the U.S., the Patient Self-Determination Act (1990) spurred hospitals to inform patients of their right to advance directives, and community programs like “Respecting Choices” have achieved high rates of ACP completion in some regions [4]. Many Western populations report positive attitudes and participation: a survey in Norway found over 90% of respondents willing to engage in ACP [5], and a UK nursing home study showed ~80% of residents had completed ACP discussions.In contrast, ACP in China is still in its nascent stages on a nationwide scale [6]. China has the world’s largest aging population and a rapidly growing need for end-of-life planning, yet ACP is not widely practiced or understood [7]. Until recently, most research in China focused on specific subgroups (e.g., cancer patients or healthcare workers) rather than the general public [8].China ranked 71st out of 80 countries in one 2015 global “Quality of Death” index, reflecting limited palliative care and planning services [9].

Overall, the influencing factors of the low uptake include cultural factors, healthcare providers’ knowledge and attitudes, the main stakeholders of ACP, i..e, patients and their families, as well as the communications among these stakeholders [10]. Culture profoundly shapes attitudes toward ACP and palliative care. In many Asian societies, and especially in China, traditional values influence how end-of-life decisions are made. In Chinese culture, open discussion of death is often seen as taboo -talking about death or dying is thought to invite bad luck or be emotionally harmful [3]. As a result, families may resist ACP conversations for fear of distressing elders. It is common for Chinese families to object to telling a terminally ill patient about a bad diagnosis or prognosis, preferring instead to “protect” the patient from upsetting news. Understanding these cultural influences is crucial for implementing ACP in China. Approaches that work in Western settings (direct, patient-led discussions) may need adjustment in China [11]. For example, involving family members early and framing ACP as a way to reduce burden on the family (rather than solely as individual rights) can resonate better with Chinese values [12].

Healthcare providers’ knowledge and attitudes toward ACP greatly influence its implementation. Research indicates that Chinese healthcare professionals generally support the principles of ACP and palliative care, but many feel inadequately prepared to practice it [12]. In a nationwide survey of 531 Chinese nurses, 92.5% of respondents agreed that ACP should be implemented, reflecting overwhelmingly positive attitudes [13]. Nurses recognize ACP as part of good end-of-life care and value its role in respecting patient wishes. They also highly value palliative care’s goals; nurses and physicians who have seen terminal suffering often appreciate interventions that improve quality of life. However, the same survey revealed significant knowledge and practice gaps: none of the nurses could answer all ACP knowledge questions correctly, and fewer than 4% had actually engaged in all the recommended ACP activities (such as initiating discussions or helping patients fill out directives) [14]. This disparity between attitude and practice suggests that while providers conceptually endorse ACP, they lack training, confidence, or institutional support to carry it out. Overall, Chinese healthcare providers value ACP’s potential to improve end-of-life decision-making, but they cite the need for better knowledge, clear guidelines, and support from their institutions. The positive attitude is a strength to build on: if given the tools and a supportive framework, many nurses and doctors are willing to champion ACP in their practice.

Patients and families are the ultimate stakeholders of ACP, and their perceptions determine whether ACP is embraced or avoided [15]. In many cases, patients and families have misunderstandings or mixed feelings about ACP. A common misconception is equating ACP (or palliative care) with “giving up” on treatment. For instance, some patients believe that choosing comfort care or drafting an advance directive means their doctors and family are abandoning hope or will stop all care [16]. In China, it is frequently the family (adult children) who drive or delay decisions. Research has shown that families sometimes make decisions counter to a patient’s stated ACP if they believe it’s in the patient’s best interest or aligns with filial obligations (for example, insisting on feeding tube insertion despite the patient’s prior refusal, as in some well-known cases) [17]. This means that even if a patient has an advance plan, family acceptance is crucial in the Chinese context.

Another factor is lack of knowledge and communication. Patients who have never heard of ACP or do not understand it will naturally not see its relevance. Until recently, the concept of ACP was poorly recognized among the general Chinese population [18]. When individuals don’t know that they have the option to plan ahead, or what benefits it brings, they cannot form positive attitudes toward it. Misinformation also plays a role: some may conflate ACP with euthanasia or think signing an advance directive might lead doctors to withdraw all treatment, reflecting a distrust or fear of the healthcare system’s intentions. In settings where doctor-patient trust is fragile, patients might suspect that a doctor suggesting ACP is trying to ration care or avoid responsibility. All these negative perceptions, such as loss of hope, upsetting the family, distrust in the system, or fatalism, contribute to low uptake of ACP.

While the body of research on ACP and palliative care has grown, important gaps and inconsistencies remain, particularly regarding the alignment of healthcare providers’ perspectives with those of patients and families. One major gap is the disconnect between what providers assume and what patients/families actually want or understand [19]. Healthcare professionals may underestimate patients’ openness to ACP due to cultural stereotypes, or conversely, they might overestimate patients’ understanding of their illness and options. While, the integration of family in ACP discussions is another area needing more exploration. Literature globally is increasingly acknowledging that ACP should sometimes be “advance care planning” for the family unit, not just the individual patient [20]. However, some Chinese clinicians might assume “families will never agree to ACP” and therefore avoid the topic, while studies of Chinese patients show that many would accept ACP if approached with sensitivity. This misalignment suggests a need for research that directly compares and mediates these viewpoints.

Another gap lies in the evidence on culturally tailored interventions. Most ACP intervention research comes from Western countries. There is still a shortage of rigorous trials or implementation studies in the Chinese healthcare context. For instance, we lack data on which specific communication techniques best increase ACP uptake among various Chinese populations (urban vs rural, educated vs less educated, etc.). Moreover, existing literature often identifies problems (lack of awareness, training needs, cultural barriers) but less often provides evidence-based solutions specific to the Chinese context [21].

Based on the research gaps mentioned above, this research combined a cross-sectional survey with qualitative interviews to measure knowledge, attitudes, and practices (KAP) regarding ACP as well as perceived barriers among nurses, patients, and family members, aiming to bridge the gap between healthcare providers and patients/families in ACP, while contributing to culturally sensitive, evidence-driven strategies.

## 2. Methods

### 2.1. Participants

This study employs a mixed-method design combining a cross-sectional survey with qualitative interviews to assess the knowledge, attitudes, and practices (KAP) regarding advanced care planning (ACP), as well as perceived barriers among key stakeholders. Participants include healthcare professionals, patients, and family members. This study was approved by the Ethics Committee of Lishui Central Hospital (Ethics Approval No. [2023] 743) on 7^th^, Dec, 2023. All participants ticked the informed consent form on the first page of the questionnaire; only those who confirmed their consent by checking the box were allowed to continue with the survey. For healthcare professionals, convenience sampling were used to select frontline nursing staff working in inpatient wards in Lishui Hospital. Inclusion criteria for healthcare professionals are ①holding a valid nursing license; ②full-time frontline nursing staff working in inpatient wards in Lishui Hospital, and ③volunteered to provide informed consent, while those not working in inpatient wards (e.g., in disinfection supply centers, intravenous medication preparation centers) and nursing students or trainee nurses were excluded. In addition, information on whether participants had training/experience in palliative care and their specific experience in communication was collected to allow subgroup analyses.

For patients, convenience sampling were applied to recruit hospitalized individuals eligible for ACP discussions who can communicate effectively and provide informed consent, excluding those with severe cognitive impairments or in critical care settings. Family members, defined as the primary caregivers or decision-makers for the selected patients, were recruited using similar criteria. The research data collection commenced on 15^th^ January, 2024, and concluded on 15^th^ December 2024. By the conclusion of the study, a total of 2,184 questionnaires were collected. After excluding incomplete responses, 2,084 fully completed questionnaires were retained for analysis, comprising 843 from nurses, 384 from patients, and 857 from patients’ family members.

### 2.2. Questionnaires and semi-structured interview

A mixed-method design was adopted to capture a comprehensive picture of ACP implementation. The quantitative component involved a cross-sectional survey, while the qualitative component consisted of semi-structured interviews and focus group discussions. This dual approach allowed for the integration of statistical data with rich, contextual insights regarding the experiences and perceptions of different stakeholders.

In specific, three tailored questionnaires were developed for nurses, patients, and family members, respectively. The questionnaire for nurses assessed their knowledge, attitudes, practices, and perceived barriers regarding ACP (see S1 File: Questionnaire). For these nurses, demographic items (e.g., age, professional role, years of experience, department) were included, and familiarity with ACP concepts, guidelines, and training was assessed. Attitudes were measured using Likert-scale items regarding the importance and effectiveness of ACP, current practices such as ACP discussions and documentation were evaluated, and open-ended questions were incorporated to identify institutional, cultural, and resource-related obstacles.

For patients and their family members, demographic and health status information was gathered, their understanding of ACP, attitudes and willingness to engage in ACP discussions were evaluated via both questionnaires (See S2 File: Questionnaire) and a random semi-structured interview. The interview guide was developed based on the multivariable regression analysis we conducted based on the findings from the nurses’ questionnaire as well as a comprehensive literature review identifying critical gaps in existing research on Advance Care Planning (ACP) within the Chinese healthcare context [22]. Specifically, the guide aimed to explore the discrepancies between healthcare providers’ and patients/family members’ perceptions and attitudes toward ACP, identify cultural influences, barriers to acceptance, informational needs, and preferences regarding healthcare communication.

Initially, domains and themes were extracted from existing literature to inform the structure of the interview questions. Major domains included (1) general understanding and awareness of ACP, (2) personal attitudes toward ACP, (3) cultural influences and family dynamics, (4) perceived barriers to ACP acceptance, (5) experiences and expectations regarding healthcare providers, (6) informational and educational needs, and (7) suggestions and recommendations for improving ACP practice. Within each domain, a series of open-ended questions were crafted to encourage rich, qualitative responses, enabling participants to express their personal perceptions and experiences freely and comprehensively. In total, 20 interview questions were formulated, ensuring that the guide adequately covers each research gap and theme identified.

To enhance clarity and ensure cultural appropriateness, the draft interview guide underwent an expert validation process. A panel comprising two senior palliative care clinicians, one nursing educator, and one qualitative research expert reviewed the questions for relevance, sensitivity, and comprehensibility. Feedback from the expert panel resulted in minor modifications to the wording of specific questions to align more closely with cultural sensitivities and communication norms.

Finally, a pilot test was conducted with three individuals representing the intended study population (patients and family members). Feedback from this pilot confirmed the clarity, comprehensibility, and appropriateness of questions, and no further substantial revisions were deemed necessary. The finalized semi-structured interview guide was then prepared for data collection in the subsequent phases of this research study.

### 2.3. Data collection

#### 2.3.1. Quantitative data collection.

Data were collected via electronic questionnaires created on the Wenjuanxing platform(The specific results are presented in S3–S5 Files). The survey link and QR code were distributed by the nursing directors of the hospital, who forwarded the information to head nurses, who then disseminated it to the target groups. All questionnaires included a standardized set of instructions that explained the study’s purpose, the nature of the research, and the requirement for informed consent. Each questionnaire was designed with mandatory responses for every item, and duplicate submissions from the same IP address were prevented.

The nurse questionnaire included demographic variables such as age, gender, marital status, religious beliefs, educational level, professional title, and years of work experience. Subsequently, multivariable regression analyses were conducted to examine the associations between these demographic and experiential variables and nurses’ ACP-related knowledge, attitudes, and practices (KAP) scores, with the significance level set at p < 0.05. The findings are presented in Table 1.

**Table 1:** Multivariable regression analysis of factors associated with ACP-KAP scores among nurses (N = 843).

Variable	Comparison (Reference Group)	β Coefficient	95% Confidence Interval	Significance
**Age**	<31 vs. ≥ 31	0.48	(−0.67, 1.60)	NS
**Gender**	Male vs. Female	1.4	(−0.71, 3.40)	NS
**Marital Status**				NS
Unmarried	vs. Divorced (Reference)	−3.8	(−11.0, 3.00)	NS
Married	vs. Divorced (Reference)	−2.2	(−9.00, 4.60)	NS
**Religious Beliefs**				NS
Buddhism	vs. Taoism (Reference)	−6	(−23.0, 11.0)	NS
**Clinical Work Experience**	≥9 years vs. < 9 years (Reference)	0.27	(−0.88, 1.40)	NS
**Educational Background**				Marginal
College	vs. Undergraduate (Reference)	1.4	(−0.19, 3.00)	NS
Master’s and Above	vs. Undergraduate (Reference)	1.2	(−0.81, 3.30)	NS
**Professional Title**				
Senior	vs. Junior (Reference)	3.5	(0.35, 6.60)	**Significant**
Intermediate	vs. Junior (Reference)	0	(−1.30, 1.30)	NS
**Number of Terminally Ill Patients**				
6–10 cases	vs. ≤ 5 cases (Reference)	3.9	(1.90, 6.00)	**Significant**
11–15 cases	vs. ≤ 5 cases (Reference)	0.54	(−6.20, 7.30)	NS
≥ 16 cases	vs. ≤ 5 cases (Reference)	3.3	(−0.40, 6.90)	Borderline
**Willingness to Promote ACP**	Willing vs. Not willing (Reference)	5.4	(2.00, 8.70)	**Significant**

Note: NS* *=* *not significant (p* *≥* *0.05); Borderline* *=* *approaching significance; Marginal* *=* *overall effect approaches significance. The original data can be found from S3 File.

The findings from these analyses highlight the impact of professional seniority, clinical experience with terminally ill patients, and willingness to promote ACP on overall KAP scores. These results informed the development of a comprehensive theoretical framework, which also served as the basis for designing subsequent semi-structured interviews with patients and their families. The internal consistency reliability of the Nurses’ ACP-KAP questionnaire was evaluated using Cronbach’s alpha for the total scale and for each subscale (Knowledge and Understanding, Attitudes Toward ACP, Perceived Barriers to ACP Implementation, Current ACP Practice, and Suggestions for Improving ACP Practice). Negatively worded items were reverse-coded prior to analysis. A Cronbach’s alpha coefficient ≥0.70 was considered acceptable, ≥ 0.80 good, and ≥0.90 excellent.

#### 2.3.2. Qualitative data collection.

Based on the reply of the patients in the questionnaire, 15 patients and 20 family members (n = 35) who hold negative views ACP importance and implementation were invited for semi-structured interview, aiming to explore in depth their perceived barriers regarding ACP. An interview guide was developed based on the research objectives. Researchers further studied the data by thoroughly reading and re-reading the interview transcripts, thus establishing a deep familiarity with the content and contextual nuances of participants’ responses. Interviews were audio-recorded, transcribed verbatim, and then analyzed using Colaizzi’s 7-step method. All participants provided consent for these transcripts to be published, and the transcripts do not contain any potentially identifying information. Then, we synthesized all themes into a comprehensive narrative that detailed the full spectrum of experiences and attitudes toward ACP. Finally, the validity of the findings was enhanced by a process of member checking, whereby the preliminary interpretations and themes were returned to the participants for verification.

## 3. Data analysis and results

From the result of multivariable regression analysis (Table 1), significant positive associations with higher ACP-KAP scores were identified for professional title, clinical experience, and willingness to promote ACP. Specifically, nurses with senior professional titles had significantly higher ACP-KAP scores compared to those with junior titles (*β* = 3.50, 95% CI: 0.35–6.60, *p *< 0.05). Intermediate professional titles showed no significant difference. Nurses caring for 6–10 terminally ill patients in the past 3 months exhibited significantly higher ACP-KAP scores compared to those caring for ≤5 cases (*β* = 3.90, 95% CI: 1.90–6.00, *p* < 0.05). Nurses caring for ≥16 cases showed borderline significance. Nurses who reported willingness to promote ACP showed significantly higher ACP-KAP scores compared to those who were not willing (*β* = 5.40, 95% CI: 2.00–8.70, *p *< 0.05). Variables such as age, gender, marital status, religious beliefs, clinical work experience duration, and educational background did not show statistically significant associations with ACP-KAP scores. Overall, these findings suggest that professional seniority, increased practical exposure to terminally ill patients, and a willingness to advocate for ACP are positively correlated with nurses’ ACP knowledge, attitudes, and practices. We hypothesize that these factors are closely linked to effective communication with patients. This is a reasonable hypothesis, as nurses with greater professional experience, more frequent exposure to terminally ill patients, and a proactive approach to promoting ACP are likely to engage more extensively in patient communication during their daily practices [23].

The quantitative findings (see S4 Files. Quantitative Dataset from Nurses’ Questionnaire) from the nurse questionnaire and multivariable regression analyses indicate that nurses generally exhibit high levels of knowledge, positive attitudes, and effective practices regarding Advance Care Planning (ACP), with factors such as professional seniority, increased exposure to terminally ill patients, and a proactive willingness to promote ACP showing significant positive associations with higher ACP KAP scores. Despite these encouraging results, our qualitative analysis, which was conducted using Colaizzi’s 7-step method on the interview transcripts with patients and their family members, revealed a critical gap. Specifically, while nurses demonstrate high awareness of ACP principles, patients and their families frequently harbor misconceptions about ACP.

This divergence suggests that even though nurses are well-versed in ACP, the effective transmission of this knowledge to patients is not occurring to the same extent. Analysis of responses from the patient questionnaire indicates that patients often misunderstand the intent and scope of ACP, associating it with a loss of hope or misinterpreting it as a sign of imminent abandonment of care (**Table 2**). This contrasts with the nurse perspective, where high ACP KAP scores were observed, highlighting an apparent communication gap between providers and recipients of care.

**Table 2:** Patient perceptions of ACP importance and implementation (n = 384).

Statement	Strongly Agree (%)	Agree (%)	Neutral (%)	Disagree (%)	Strongly Disagree (%)
1. I believe that discussing my future medical care is important for planning my treatment.	30	40	15	10	5
2. ACP discussions help me feel more secure about my healthcare decisions.	25	35	20	15	5
3. I feel that engaging in ACP means my doctors are giving up on me.	20	30	25	15	10
4. I worry that ACP discussions might reduce my hope for recovery.	22	28	20	18	12
5. I perceive ACP as a process that ensures my treatment aligns with my personal values.	35	40	15	7	3

Note: the original dataset can be found from S5 File. Quantitative Dataset from patients’ and family’s Questionnaire

These data suggest that while a large majority of patients appreciate the concept of ACP, there is considerable skepticism regarding its real-world execution. Nurses demonstrated a comprehensive understanding of ACP, citing formal training and continuous professional development as key contributors. In contrast, many patients and family members had limited and sometimes inaccurate knowledge about ACP. Some patients misunderstood ACP as a way to hasten death, while family members reported that they had received little information about the benefits and goals of ACP. According to Table 2, there are 15 patients who are disagree with the significance of ACP and their family members held the same view. Therefore, we invited this group of stakeholders to the interview. The semi-structured interview further pointed out that although nurses were optimistic about ACP’s potential, the patients and family members viewed ACP as a precursor to treatment abandonment. Family members frequently echoed these sentiments, with some expressing that the concept of ACP conflicted with traditional beliefs about fighting illness until the very end. Both patients and family members expressed a desire for clearer communication, better educational resources, and more personalized approaches to end-of-life care planning. They suggested that healthcare providers should deliver personalized, empathetic information regarding ACP to alleviate fears and clarify misunderstandings. There was a consensus that multi-channel educational programs and community outreach could play a vital role in reshaping public perceptions of ACP.

## 4. Discussion

ACP has emerged as a pivotal element of palliative care, and in many countries, it has been integrated into standard clinical practice, demonstrating improved patient outcomes and satisfaction [24]. However, in China, traditional cultural values and a longstanding emphasis on aggressive treatment have contributed to widespread misunderstandings regarding ACP. In particular, many patients and their families often interpret ACP as a signal of giving up on treatment rather than as a proactive measure to ensure dignity and comfort during the terminal phase of illness. It is important to note that previous findings have been derived from separate samples across various regions or hospitals, suggesting that observed discrepancies may reflect inter-study differences rather than actual variations in clinical practice. This study is the first to address the issue within a single hospital, concurrently assessing the perspectives of both nurses and patients to provide a more integrated understanding of ACP perceptions.

In this research, while nurses report that ACP practices are well established and effective, many patients view these practices with skepticism and concern. Family members, who are often key decision-makers in healthcare, sometimes oppose ACP due to fears that it may compromise the opportunity for curative treatment. This corroborates the findings in reference, which suggests that a significant gap exists between healthcare providers’ views and those of patients and their families regarding ACP [25]. For example, previous studies have highlighted that healthcare professionals often overestimate the adequacy of ACP implementation relative to patient-reported experiences.

Moreover, studies indicate that insufficient communication and a lack of comprehensive educational programs contribute to misconceptions about palliative care [26]. Nonetheless, in research conducted in China’s context, what is frequently ignored is that, there are also inconsistencies in how ACP is defined and measured across studies [27]. Some Chinese studies equate ACP with signing a document, while others use a broader definition (including any discussion of future preferences). This makes it hard to compare results and track progress. This study is conducted based on the definition that, ACP is a process by which individuals, even when healthy, make decisions about future medical care in case of serious illness, and communicate those preferences to family and healthcare providers. Based on this we found that in China, the rapid evolution of the healthcare system, combined with persistent traditional beliefs, poses unique challenges for ACP implementation. Misinterpretations, such as equating ACP with treatment abandonment, can hinder the uptake of palliative care services and lead to suboptimal patient outcomes. As such, understanding the underlying reasons for these divergent perspectives is crucial for designing interventions that bridge the gap between clinical practice and patient/family expectations. Nurses play a critical role in implementing ACP protocols and guiding patients through the process. Despite their high valuation of palliative care, a notable disparity exists between nurses’ perceptions and those of patients and family members. Several factors contribute to healthcare providers’ stance on ACP.

First, there is the issue of education: formal training in palliative care and ACP communication has been limited in Chinese medical and nursing curricula [28]. Many providers are unsure about the legalities and proper procedures, for instance, nurses in one study found the concept of advance directives confusing and were unclear about how binding or revisable such documents are [29]. Additionally, Chinese providers operate in a system without clear ACP policies (no laws mandating recognition of advance directives), leading to role ambiguity and uncertainty about their authority to honor or discuss ACP [1]. Qualitative reports show that nurses feel ACP is part of their professional duty, but they are uncertain about how it fits within existing laws and hospital protocols [30]. Prognostic uncertainty is another hurdle: doctors and nurses may hesitate to initiate ACP if they are unsure about a patient’s trajectory or fear “taking away hope” too early [1]. Some providers also perceive that Chinese families are not ready for these conversations, and thus they hold back, creating a self-fulfilling gap [3].

The present study was designed to address these issues by comprehensively assessing ACP implementation from three distinct perspectives: nurses, patients, and family members. By employing both quantitative and qualitative methodologies, this research seeks to illuminate the barriers to effective ACP implementation and propose strategies to enhance public and professional understanding of palliative care in China. The findings of this study highlight a critical disconnect between healthcare providers’ perceptions and those of patients and their families regarding ACP implementation. Nurses, as frontline providers, reported that ACP is highly important and that current practices are generally well established. Their confidence appears to be rooted in formal training, institutional protocols, and direct clinical experience with palliative care. In contrast, patients and family members harbor significant reservations about ACP, with many perceiving it as synonymous with treatment abandonment.

### 4.1. Divergent perceptions: Nurses vs. patients and family

In terms of implementation, the discrepancy in perspectives between nurses and patients/family members is notable. Nearly all nurses endorsed the importance of ACP, and a majority felt that their institutions had implemented adequate ACP measures. However, only 30% of patients perceived that ACP was effectively implemented, and almost half of the family members believed that opting for ACP would lead to a withdrawal from active treatment. This disparity may be partly explained by differences in information sources and personal experiences. Nurses have access to professional training and up-to-date clinical guidelines, which contribute to a positive view of ACP. Meanwhile, patients and family members, lacking comprehensive education on palliative care, are more likely to rely on traditional cultural beliefs and anecdotal evidence that equate ACP with giving up on treatment.

### 4.2. Impact of information sources and education

The multivariable analysis from the nurse group (Table 1) indicates that exposure to diversified information channels, such as training from experienced colleagues, scientific publications, and media, has a positive impact on ACP KAP scores. Although similar quantitative data were not collected for patients and family members regarding their information sources, qualitative interviews revealed that insufficient and fragmented educational efforts contribute significantly to their misconceptions. This indicates the need for broader, multi-level educational initiatives that target not only healthcare professionals but also patients and their families.

Studies have found that when ACP is explained as planning for “peace of mind” or “ensuring dignity,” patients are more receptive, and families appreciate having guidance when tough decisions arise [31]. In China, recent surveys show a moderately positive acceptance level of ACP among the public when asked hypothetically. People do value a “good death” and not being a burden on family, which ACP can facilitate. The task ahead is to correct misconceptions, making clear that ACP is about preserving control and hope, not taking it away, and to provide supportive environments for these conversations so that patients and families can see ACP as a normal, even beneficial, part of healthcare planning rather than a frightening last resort.

### 4.3. Cultural context and traditional beliefs

Traditional Chinese culture often places a high value on aggressive treatment and “fighting” illness [32]. In this context, ACP may be misunderstood as a concession to terminal decline rather than a patient-centered approach that emphasizes quality of life. Our data indicate that many patients and family members remain skeptical of ACP because it is perceived as a relinquishment of hope. This cultural context must be taken into account when designing educational programs and communication strategies. Healthcare providers should not only present the clinical benefits of ACP but also engage with patients and families in a culturally sensitive manner that addresses deeply held beliefs and fears.

### 4.4. Reliability analysis of the nurses’ ACP-KAP questionnaire

The reliability analysis (Table 3) revealed substantial differences across the subscales of the Nurses’ ACP-KAP questionnaire. While the overall 25-item instrument demonstrated good internal consistency (Cronbach’s α = 0.804), the psychometric performance of the individual subscales varied considerably. The Support subscale exhibited excellent reliability (α = 0.960), indicating that items addressing institutional and policy-level support were highly consistent and measured a common construct. In contrast, the Practice subscale showed only poor reliability (α = 0.543), suggesting moderate coherence among items and the need for further refinement. More importantly, the Knowledge (α = –0.426) and Attitude (α = 0.192) subscales demonstrated unacceptable internal consistency. This finding implies that these items may not represent a unidimensional construct, possibly due to heterogeneity in item wording, the inclusion of both barrier- and value-oriented statements, or potential misalignment in the intended measurement domain.

**Table 3:** Reliability analysis of the Nurses’ ACP-KAP questionnaire.

Questionnaire/ Subscale	Number of items	Cronbach’s α	Average inter-item correlation
Knowledge (Q1–Q8)	7	−0.426	−0.049
Attitude (Q9–Q15)	7	0.192	0.035
Practice (Q16–Q20)	5	0.543	0.097
Support (Q21–Q25)	5	0.96	0.827
Total KAP (Q1–Q20)	19	0.348	0.014
Total (Q1–Q25)	24	0.804	0.078

As shown in Table 3, internal consistency varied substantially across subscales. The Support subscale demonstrated excellent reliability (α = 0.960), while Practice showed poor-to-moderate consistency (α = 0.543). In contrast, both Knowledge (α = −0.426 after excluding an aberrant item) and Attitude (α = 0.192) exhibited unacceptable internal consistency. For the overall instrument, reliability was good when all 25 items were included (α = 0.804), but unacceptable when restricted to the ACP-KAP items (Q1-Q20; α = 0.348), indicating that the supportive/organizational items (Q21-Q25) substantially contribute to the scale’s internal consistency.

These results highlight that, although the full questionnaire can be used to capture a general assessment of ACP-related knowledge, attitudes, practices, and supportive environments, the independent use of the Knowledge and Attitude subscales is problematic. Future studies should therefore consider revising and revalidating these domains, for example by refining item content, ensuring consistent response scaling, and conducting exploratory or confirmatory factor analyses to verify dimensionality. Such modifications will be essential to enhance the psychometric robustness of the instrument and to ensure valid and reliable measurement of nurses’ ACP-related knowledge and attitudes.

### 4.5. Implications for clinical practice and policy

The divergent perceptions identified in this study have significant implications for both clinical practice and healthcare policy. First, there is a clear need to bridge the gap between provider and patient/family perspectives. Establishing a standard, culturally relevant definition of what constitutes “ACP completion” in China (perhaps one that counts documented discussions in medical records even if no legal form exists) would help unify research efforts.

Hospitals and clinical institutions should prioritize enhancing communication strategies that help patients and family members understand the true purpose of ACP. This could involve specialized training for nurses in empathetic communication, the development of patient-friendly educational materials, and the integration of ACP discussions into routine care in a way that is both informative and supportive. Second, policy-makers should consider the establishment of standardized protocols for ACP that include mandatory educational components for patients and family members. By ensuring that the public receives consistent, evidence-based information about ACP, it may be possible to shift cultural perceptions and increase acceptance of palliative care practices. Third, future research should explore the effectiveness of multi-channel education interventions that involve community outreach, media campaigns, and interactive sessions with healthcare providers. Such studies could assess whether improved public understanding of ACP leads to greater acceptance and more effective implementation in clinical settings.

## 5. Conclusion

In summary, our study highlights a significant gap between the perspectives of nurses and those of patients and family members regarding the implementation of ACP in a Chinese hospital setting. While nurses, supported by formal training and clinical experience, report a high level of confidence in the current ACP practices, many patients and family members remain unconvinced. They often view ACP as tantamount to abandoning treatment, reflecting a deep-seated cultural bias and a lack of comprehensive, accessible education on palliative care. The findings indicate that although the theoretical importance of ACP is widely recognized, its practical application falls short in the eyes of patients and families. This divergence highlight the need for targeted interventions that focus on improving communication strategies, enhancing public and patient education, and ultimately, aligning clinical practices with the expectations and needs of patients and their caregivers. To move forward, healthcare institutions must adopt a more patient-centered approach, ensuring that ACP discussions are conducted in a manner that is both empathetic and informative. Additionally, policy-makers should consider developing standardized protocols that mandate robust educational components as part of the ACP process.

In China, family involvement is a given, yet guidelines on how to involve them without marginalizing the patient’s voice are not well developed. Research could focus on models of shared decision-making that respect both patient autonomy and family values.

## 6. Strengths and limitations

This study has several strengths. The mixed-method approach enabled a comprehensive assessment of ACP from multiple perspectives, combining the breadth of quantitative data with the depth of qualitative insights. The use of tailored questionnaires ensured that data were collected on the most relevant aspects for each stakeholder group. To the best of our knowledge, this is the first study conducted within a single hospital that explores the diverse attitudes of different stakeholders toward this important issue.

However, there are limitations to consider. The use of convenience sampling may limit the generalizability of the findings to other settings or regions in China. Additionally, the cross-sectional nature of the survey does not allow for causal inferences. Future studies should employ longitudinal designs to better understand the evolving perceptions of ACP and the impact of targeted interventions over time. Moreover, future studies should incorporate detailed pathology and disease trajectory data, as variations in illness progression may significantly shape the timing, content, and receptivity of anticipatory decision-making processes in ACP.

## Supporting information

S1 File Nurses’ Advance Care Planning (ACP) questionnaire. (DOCX)

S2 File Patients’ Advance Care Planning (ACP) questionnaire. (DOCX)

S3 File Qualitative data from nurses’ questionnaire. (XLSX)

S4 File Quantitative dataset from nurses’ questionnaire. (XLSX)

S5 File Quantitative dataset from patients’ and family’s questionnaire. (XLSX)
