# Supplementary material for: Nurses’ high valuation of palliative care versus patient and family misconceptions: A mixed-approach study of Advanced Care Planning implementation in China
Source: PLoS One. 2025 Oct 7;20(10):e0333739. doi: 10.1371/journal.pone.0333739 (PMC12503265; doi:10.1371/journal.pone.0333739)
Supplement: S1 File — (DOCX) [file pone.0333739.s001.docx]

### Appendix I ****Nurses’ Advance Care Planning (ACP) Questionnaire****

**Instructions:**
Please rate the following statements based on your level of agreement:

**Scale:**
1 = **Strongly Disagree** ; 2 = **Disagree**; 3 = **Neutral**; 4 = **Agree**; 5 = **Strongly Agree**

### ****Knowledge and Understanding of ACP****

1. I clearly understand the concept of Advance Care Planning (ACP).
2. I know the differences between ACP and advance directives (e.g., living wills, DNR orders).
3. I am familiar with the current legal status of ACP and advance directives in China.
4. I understand how binding advance directives are in clinical practice.
5. I know clearly how ACP fits within my professional duties according to existing hospital policies.

### ****Attitudes Toward ACP****

1. ACP discussions are essential components of quality patient care.
2. It is beneficial to initiate ACP conversations early, before critical illness occurs.
3. I feel comfortable discussing ACP with patients and families.
4. Discussing ACP could make patients and families lose hope. (reverse-coded)
5. ACP discussions can positively influence patient autonomy and decision-making.

### ****Perceived Barriers to ACP Implementation****

1. Limited formal education in ACP prevents me from initiating ACP discussions confidently.
2. Uncertainty about ACP-related laws and hospital policies inhibits my willingness to discuss ACP.
3. Difficulty in predicting patient prognosis makes me hesitant to start ACP conversations.
4. I perceive that most patients and families in China are culturally resistant to ACP.
5. Time constraints in daily clinical practice significantly limit ACP discussions.

### ****Current ACP Practice****

(Scale: 1 = **Never**, 2 = **Rarely**, 3 = **Sometimes**, 4 = **Often**, 5 = **Always**)

1. I actively initiate ACP discussions with eligible patients.
2. I document ACP discussions and patient decisions clearly in medical records.
3. I regularly collaborate with other healthcare providers (physicians, nurses, social workers) during ACP processes.
4. I revisit ACP conversations periodically, reflecting changes in patient condition or preferences.
5. I encourage families to participate actively during ACP discussions.

### ****Suggestions for Improving ACP Practice****

(Scale: 1 = **Not Helpful**, 2 = **Slightly Helpful**, 3 = **Moderately Helpful**, 4 = **Very Helpful**, 5 = **Extremely Helpful**)

1. Formal ACP training workshops or continuing education programs
2. Clear institutional ACP policies and guidelines provided by the hospital
3. Regular interdisciplinary meetings focused on ACP discussions
4. Formal legal recognition and clearer national guidelines for ACP and advance directives
5. Inclusion of ACP training in medical and nursing school curricula

**Optional Open-ended Question:**
Please briefly describe one suggestion you have for making ACP discussions more effective or acceptable in your clinical practice:
