# Supplementary material for: Nurses’ high valuation of palliative care versus patient and family misconceptions: A mixed-approach study of Advanced Care Planning implementation in China
Source: PLoS One. 2025 Oct 7;20(10):e0333739. doi: 10.1371/journal.pone.0333739 (PMC12503265; doi:10.1371/journal.pone.0333739)
Supplement: S2 File — (DOCX) [file pone.0333739.s002.docx]

### S2 Appendix: ****Patients’ Advance Care Planning (ACP) Questionnaire****

**Questionnaire**:

**Section 1: General Understanding and Awareness of ACP**

Could you describe your understanding of Advance Care Planning (ACP)? How would you explain it in your own words?

Have you previously heard about ACP or related concepts (like advance directives, living wills, or palliative care)? If yes, where did you learn about these terms?

**Section 2: Personal Attitudes Toward ACP**

What are your thoughts about discussing future medical care decisions in advance, especially concerning serious illnesses?

How comfortable do you feel about having conversations related to end-of-life care decisions with your family or healthcare providers? Why or why not?

Do you feel ACP discussions could benefit you or your family? Could you explain your reasons?

**Section 3: Cultural Influences and Family Dynamics**

In your family, who typically makes important healthcare decisions, especially when someone is seriously ill? Can you give an example?

How do traditional Chinese values like filial piety or respecting elders influence your family’s healthcare decisions?

Do you feel ACP aligns with or conflicts with your cultural beliefs about discussing death or serious illness openly? Could you elaborate?

**Section 4: Barriers to ACP Acceptance**

What would make you hesitant or uncomfortable about participating in ACP discussions?

Some people feel that talking about ACP means giving up hope. Do you agree with this perception? Why or why not?

Are there any family dynamics or concerns that would discourage you from discussing ACP with your loved ones or healthcare providers?

Do you worry that documenting your future healthcare wishes (e.g., in an advance directive) could negatively influence how doctors treat you in the future? Why or why not?

**Section 5: Experiences and Expectations with Healthcare Providers**

Have any healthcare professionals ever initiated ACP-related conversations with you or your family members? If yes, how did the conversation go? If no, would you prefer them to initiate such conversations?

How confident are you that your healthcare providers understand or would honor your wishes regarding medical treatment if you expressed them clearly?

What qualities or communication approaches would make you feel comfortable discussing ACP with healthcare providers?

**Section 6: Information and Education Needs**

What type of information or resources would help you better understand ACP?

Would you prefer receiving information about ACP individually or in family discussions involving a healthcare professional? Could you explain your preference?

How do you feel about participating in educational sessions or workshops designed to discuss ACP? Would you attend, and what factors might influence your decision?

**Section 7: Suggestions and Recommendations**

In your opinion, what would be the best way for healthcare providers to introduce ACP to patients and families in a culturally respectful manner?

What suggestions do you have for healthcare policymakers or hospitals to make ACP discussions more acceptable and accessible to Chinese families?
